# Voicing Caregiver Experiences: Wellbeing and Recovery Narratives for Caregivers

**DOI:** 10.1192/pb.bp.114.046771

**Published:** 2015-02

**Authors:** Debbie Mountain

**Figure:**
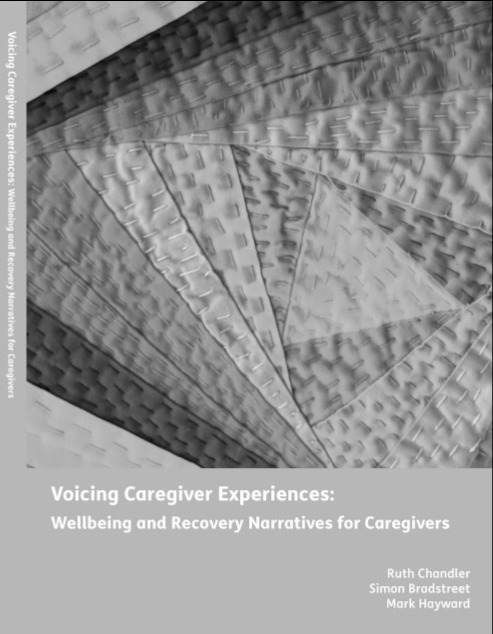


In this easy-to-read book, ten authentic carer stories of different styles have been collected. Each has different themes, many of sadness and loss, and they describe the roller-coaster ride that most have had to endure until eventually finding stability and some acceptance of the situation in their own and their loved ones’ lives. Tips between carers are shared, especially those of encouraging and steering others towards empowerment both in managing their own, often ignored, needs and to negotiate the fragmented, bewildering and inconsistent care delivery arrangements.

The most notable theme is that of hope. The stories demonstrate the process of finding hope, not a superficial denial of the challenges that lie ahead, but something worthy of respect. This hope is borne in adversity, is effortful and those who do find it have to overcome years of difficult associations to have future positive expectations.

Another theme is that of the care triangle between service users, carers and professionals. Many speak of how devastating some professional attitudes and practices can be, leaving wounds that take years to heal. Many also speak of positive relationships with professionals that have the capacity to become pillars of strength.

These stories are of heroes, and their own contribution to their relative’s wellness is often under-recognised, overlooked and underestimated. In addition, their own care needs are often ignored as their caring role becomes engulfing. Many require active encouragement and permission to attend to their own wellness. When we as professionals see carers presenting as fraught or distressed, we should honestly ask ourselves ‘What would we do?’ After reading these stories, I am doubtful any of us, even experienced clinicians, would know what it takes to manage some of the situations described, let alone find the peace than many carers achieve.

